# Future-proofing extensive livestock production in subtropical grasslands and savannas

**DOI:** 10.1093/af/vfad045

**Published:** 2023-10-13

**Authors:** Kevin P Kirkman, Richard W S Fynn, Devan McGranahan, Peter J O’Reagain, Trevor Dugmore

**Affiliations:** School of Life Sciences, University of KwaZulu-Natal, Pietermaritzburg, South Africa; Okavango Research Institute, University of Botswana, Maun, Botswana; Agricultural Research Service, United States Department of Agriculture, Miles City, Montana, USA; Queensland Department of Agriculture and Fisheries, Charters Towers, QLD 4820, Australia; Livestock Production Science, KwaZulu-Natal Department of Agriculture and Rural Development, Cedara, South Africa

**Keywords:** forage quality, grassland and savanna management, grazing strategies, heat-resistant livestock

**Implications**

- Predicted impacts of climate change will negatively affect extensive livestock production in subtropical grasslands and savannas unless proactive strategies are developed to mitigate negative impacts.
- Livestock adaptation, via breeding for future environments, is key to ensuring livestock health and performance from animals adapted to extensive grazing in hot and unpredictable environments.
- Grassland and savanna grazing management to ensure an adequate supply of the highest-quality wet season forage (quality) and adequate volumes of dry season forage (quantity) is critically important for extensive livestock production under adverse conditions.
- Breeding (small adapted animals) and feeding (wet season quality and dry season quantity) are key strategies for future-proofing livestock production in extensive conditions.

## Introduction

Grasslands and savannas cover a majority of the Earth’s surface and have been heavily impacted, fragmented, and transformed by anthropogenic activities (Buisson et al., 2021). In many parts of the world, extensive livestock production is the most sustainable agricultural option due to biophysical constraints on cropping. Well-managed livestock grazing not only directly produces animal protein but supports the delivery of a breadth of ecosystem services from grasslands (Lemaire et al., 2011).

Extensive livestock production relies upon a constant forage supply (Rust, 2019) throughout the year, with quality meeting the nutritional requirements of growing or lactating animals. In the future, managing subtropical grasslands and savannas for livestock production will be complicated by global change factors including reduced or erratic rainfall patterns and increased temperatures (Giridhar and Samiredddypalle, 2015). Managers must also cope with greater scrutiny of the environmental impacts of livestock production and animal welfare, along with concerns over biodiversity impacts and greenhouse gas emissions.

### Livestock and climate stress

The most important direct effects of increasing temperatures on livestock are reduced nutrient intake and heat stress (Mariara, 2008). Higher ambient temperature can elevate body temperature, to which cows respond by decreasing feed intake by 3% to 5% per additional degree of temperature (Collier and Gebremedhin, 2015). Heat stress increases respiration and mortality, reduces fertility, modifies animal behavior, and suppresses immune and endocrine systems, thereby increasing disease susceptibility (Silankove, 2000). At the same time, exposure to vector-borne diseases—which can be strongly influenced by climate change—and transmission of wild-borne diseases like foot and mouth disease is likely to increase under predicted climate change scenarios (Savasni et al., 2015).

Breeding and cross-breeding might improve heat tolerance, as there are substantial differences among breeds’ inability to cope with heat stress, even among high-yielding genotypes. Small bodied cattle breeds are more heat tolerant (Elayadeth-Meethal et al., 2018). The adoption of drought-tolerant ruminant livestock species and/or breeds that are capable of efficiently utilizing poor quality roughages needs to be undertaken. This would entail exploiting local or indigenous breeds of cattle, sheep, and goats (Moyo and Nsahlai, 2021).

**Figure 1.**
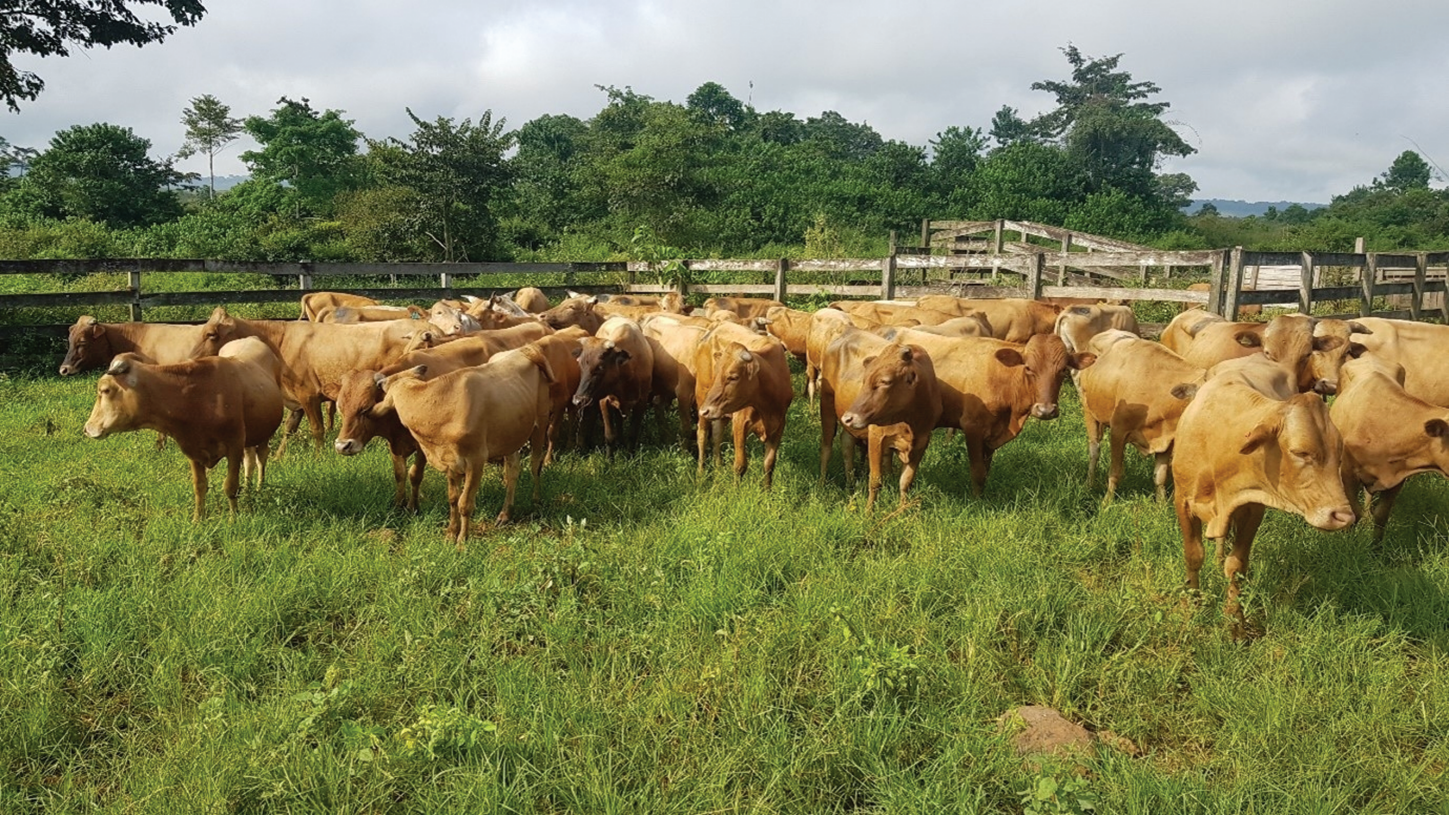
N’dama cattle in equatorial savanna in Gabon. Mature weight ~250 kg, well adapted to local forage quality, heat, and diseases. Photo by K P Kirkman.

Smaller body size of tropical indigenous cattle breeds is recognized as being beneficial for surviving in harsh environments, due, in part, to the smaller animals’ lower feed and water requirements. High heat tolerance may, in part relate, to a greater ratio of surface area to BW, (Tadessea et al., 2019) with smaller animals having a greater relative surface area to lose heat from, relative to larger animals (Figure 1).

Biological efficiencies of cow and calf to weaning or yearling weights were superior for small cows (Morris and Wilton, 1976). Indigenous small framed breeds in Namibia produced more kg weaner mass produced/100 kg cow mated than larger framed breeds (Lepen, 1996). Also in Namibia Els (2002) showed that frame size was related to productivity, when stocked at the same biological weight per ha, with small framed animals had a higher production per ha than large framed animals. Environmentally, semi-arid cattle producing regions can very effectively take advantage of the lower production cost and increased pasture-carrying capacity associated with maintaining cows of a smaller frame size that will result in greater net return per ha per cow (Senturklu et al., 2021).

**Figure 2.**
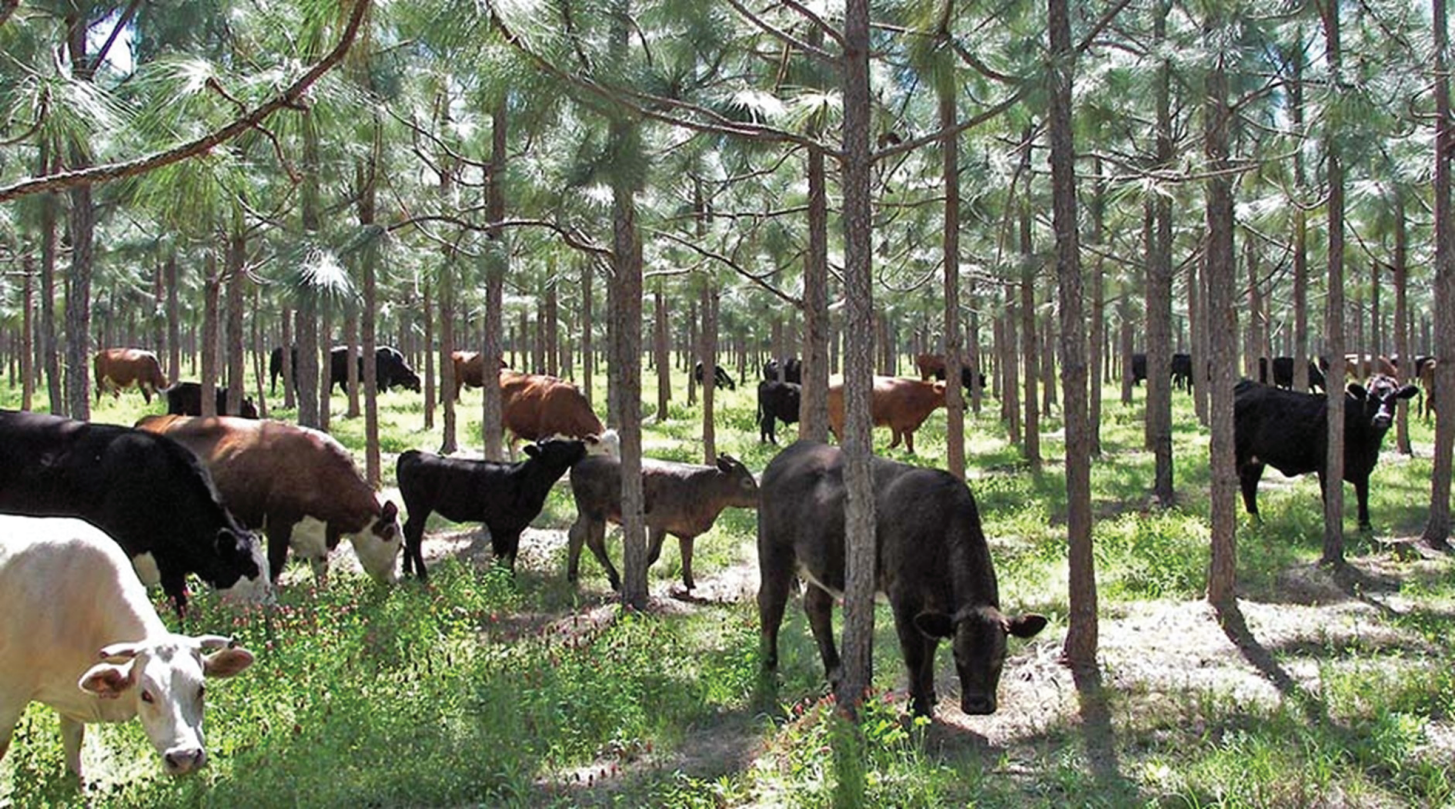
Cattle grazing in a pine plantation silvopasture in the southeastern United States. Photo by Richard Straight, USDA FS National Agroforestry Center, CC BY-NC.

Strategies to reduce the impact of hot conditions include ensuring optimal quality forage to compensate for decreased intake, reducing walking during the hottest time of the day as it increases a cow’s heat load, allowing full access to grazing at night, and providing shade throughout the grazing unit. Cows will graze up to 70% of their daily grazing at night in hot weather (Savasni et al., 2015). The shade provided by “silvopasture”—a practice in which animals graze under intentionally-managed stands of shade-providing trees that in turn deliver ecosystem services (Figure 2)—substantially reduces ambient air temperatures for livestock across Latin America and Africa (Zeppetello et al., 2022). Silvopasture also has a long history in the southeastern USA, and is undergoing a resurgence as part of regional efforts to adapt to global temperature change (Smith et al., 2022).

### Forage resources

In general, grazing natural grasslands is an economical feed source for livestock, requiring little expenditure on additional resources that would otherwise increase cost of production as well as environmental impact. Converting diverse natural agroecosystems to low-diversity, intensive crop or pasture systems can substantially reduce the delivery of supporting ecosystem services and greatly diminish soil carbon pools (Knops and Tilman, 2000).

Rainfall amount and seasonal distribution, climate, and soil influence the quantity and quality of grassland forage (Owen-Smith, 2008), which interacts with grazing effects on grassland structure and maturation (Hempson et al., 2015). In the dry season, quality decline is driven by changes between carbon assimilation and soil nutrient supply (Ellery et al., 1995).

Reduced soil nitrogen caused by more rapid plant growth over extended growing seasons in a warmer, carbon enriched environment are likely to cause a decline in forage quality (Morris et al., 2022). Increasing temperatures are further projected to reduce forage quality by lowering digestibility and crude protein content of feeds (Polley et al., 2013). For every 1 °C rise in ambient temperature, the neutral detergent fiber (NDF) content of feeds increases by 0.4% (Moyo and Nsahlai, 2021). Because NDF correlates with forage dry matter digestibility (DMD), each 1% increase in NDF can drive a 0.6% decline in DMD (Lee et al., 2017). Similarly, Moyo and Nsahlai (2021) suggest that, the rumen degradability of feeds would most likely decrease by 0.6% for every 1 °C increase in ambient temperature.

Lower-latitude regions are projected to experience greater livestock production loss than temperate regions (Moyo and Nsahlai, 2021; Thornton et al., 2022). Sub-Saharan Africa is particularly hard-hit by temperature-driven impacts on meat and milk production, while projected negative impacts on beef production in central America are also high (Thornton et al., 2022). Heat stress is consistently projected to become a serious challenge in cattle production systems through this century, leading to decreases in milk and meat production (Thornton et al., 2022).

### Grassland and savanna management

The livestock heat stress and health issues outlined above require targeted interventions that are likely to involve adapting livestock via breeding for future scenarios. Equally important is ensuring a constant, high-quality supply of forage in a sustainable manner without degrading the resource and lowering productive capacity as has happened in many areas of the world.

As such, managers must simultaneously consider both the grassland resource (supply) as well as the livestock (demand), which can impact both the livestock production enterprise as well as the health of the grassland ecosystem. Grassland management options include the use of fire (Fuhlendorf et al., 2017) and the manipulation of livestock grazing patterns and intensity. In the case of livestock manipulation, variables for manipulation include livestock type, livestock numbers (stocking rate), livestock density (spatial distribution), and livestock movement (intra- and interseasonal movement or temporal distribution), in which density and movement are coupled. Stocking rate influences livestock performance (Jones and Sandland, 1974) as well as the impact of grazing on the ecosystem (Gibson, 2009), with excessive stocking rates having the potential to lead to desertification and loss of productive capacity. Manipulating livestock density involves restricting livestock to grazing specific areas at higher density for various periods of time, with corresponding periods of absence in the grazing cycle. Interseasonal movement can influence supply of forage through the year, while intraseasonal movement influences the quality and quantity of intake. Both inter- and intraseasonal movement have the potential to influence defoliation patterns, vegetation structure and maturity, and selectivity on the part of the grazers, which in turn may influence livestock performance as well as livestock impact on the grassland ecosystem (Briske et al., 2008; Teague et al., 2013).

**Figure 3.**
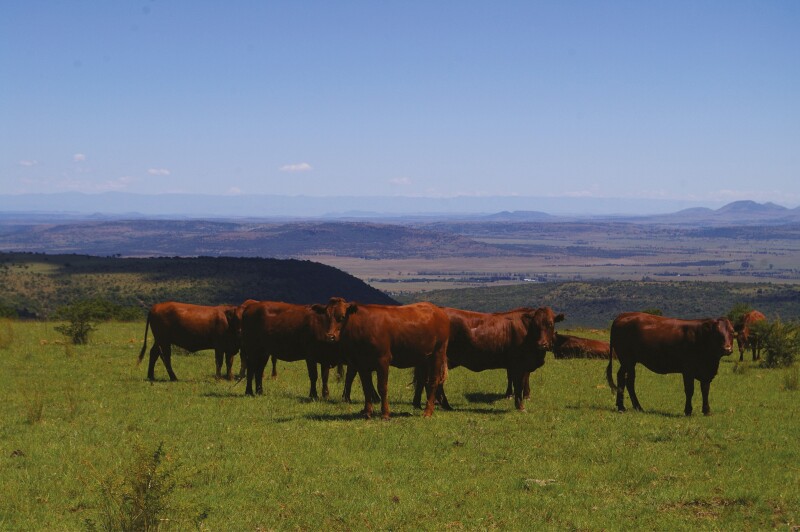
Cattle concentrated on a wet season priority paddock in a flexible grazing management system in a mesic grassland. Photo by K P Kirkman.

**Figure 4.**
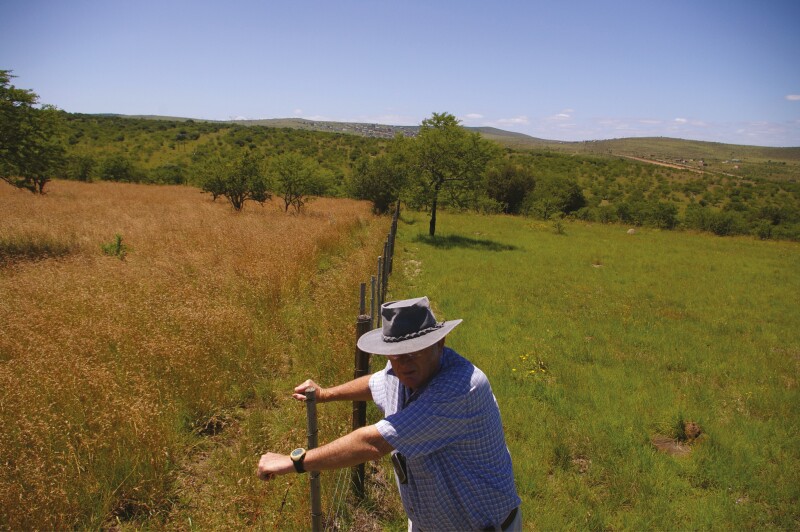
Wet season grazing on the right of the fence, and rested grassland on the left of the fence in preparation for dry season grazing. Photo by K P Kirkman.

A key constraint for animal performance, in mesic grasslands especially, is the decline in forage quality as grassland increases in biomass and matures during the growing season. To address this constraint, Venter and Drewes (1969) presented a flexible or open rotation grazing management system, incorporating fire, periodic full season rest, and flexible movement of livestock through variable numbers of paddocks utilized, in an attempt to maintain grass in a short, high-quality state during the summer growing season. In years of abundant growth, fewer paddocks may be used to maintain short, high-quality forage (Figure 3), while in dry years with poor growth, more or all paddocks in the planned grazing cycle are used. When livestock are returned to a paddock is based on the grass growth stage rather than time or a rigid cycle. A proportion of paddocks (priority paddocks) will be repeatedly heavily grazed in a nonselective manner (facilitated by burning in the spring where necessary to ensure all grass species start in a short, immature, and high-quality state), maintaining high quality. A proportion of paddocks (in the planned grazing cycle) will be lightly grazed in a more selective manner (depending on rainfall and growth patterns during the season). Unused (have come through a full wet season rest) or lightly grazed paddocks may be prioritized for burning, while those heavily grazed are prioritized for full season rest. They also highlight that, because a large proportion of the paddocks are grazed short and nonselectively, the requirement for fire to remove unpalatable, ungrazed grass is reduced. While not specifically highlighted originally, periodic full growing season rests have a positive impact on grass vigour, Kirkman (2002) while at the same time providing forage for winter use (Kirkman and Moore, 1995; Figure 4). While the specific system proposed by Venter and Drews (1969) has seen little adoption or reference in published literature, the general concept of adaptive, multipaddock grazing systems has been widely embraced (Teague et al. 2013).

The role of periodic, full season rests is not often adequately described in various meta-analyses and evaluations of grazing systems, probably because most management focuses on grazer movements within a single grazing season, rather than across years (Barnes, 1992; O’Reagain and Turner, 1992; Briske et al., 2008; Teague et al., 2013; Hawkins, 2017; McDonald et al., 2019). Evaluating the impacts of periodic full season rests on grassland ecosystem health and livestock performance logically requires a multiyear study, which might explain the lack of clear differentiation between the outcomes of experiments examining continuous and rotational grazing.

### Provision of forage throughout the year

Many grazing systems have only a minor focus on providing winter forage, particularly where the provision of alternate forage sources such as pastures and the preservation of forage in the form of silage or hay is economically viable. Hence early grazing systems were focused mainly on utilization of grassland during the wet season (intraseasonal) and mostly ignore interseasonal movement, apart from some recommendations for resting a proportion of grassland either for a period within a growing season or for a full growing season. This was particularly true in mesic areas where winter grass quality is typically inadequate for livestock maintenance in the absence of some form of supplementation (Kirkman and Moore, 1995). However, even in more arid regions where winter grass quality can maintain livestock condition, grazing system recommendations sometimes largely ignore the requirement for wet season resting to provide winter forage.

Many grazing management strategies ignore the different impacts that grazing has on grassland during the active growing season (summer) and the dormant season (winter). In general, summer grazing has been shown to impact the vigour and regrowth potential of the more palatable (more frequently and intensively grazed grasses) to a greater degree than the less palatable grass species which are grazed less frequently and less intensively (Barnes and Dempsey, 1992; Kirkman, 2002). Apart from any direct impacts on individual grass tufts, this influences the competitive interactions of the multispecies grass sward during regrowth (Fynn et al., 2005). Tufts that are less severely grazed and have some photosynthetic material remaining are able to regrow faster, and consequently are able to commandeer resources (nutrients, water, and light), giving them a competitive advantage. This creates a self-reinforcing feedback, which, if not interrupted, can result in rapid species composition change tending towards the less palatable, less preferred species with grazing pressure on the remnant palatable species consequently increasing rapidly.

Grazing during the dormant season essentially comprises removal of senesced material, and provided defoliation levels are not excessive, has negligible negative impact on regrowth at the beginning of the following growing season, as old senesced leaves are not photosynthetically active. Dormant season grazing has been reported to have no detrimental effect on production, cover or seed production (Rethman et al., 1971) while positive impacts may be realized by reduced shading, although few studies report explicitly on the impacts of winter grazing. These seasonal differences in grazing impacts imply that grazing management should also vary seasonally.

Forage quality and quantity (standing biomass) are usually inversely related during the growing season (Fuhlendorf et al., 2017). Short, rapidly growing grass is highly palatable, nutritious and easily digestible. Tall, slowly growing or senescing grass (typically at or after flowering stage) has a lower concentration of nutrients and higher fiber content, resulting in lower palatability and quality (Poppi, 2011). During the dormant season, the quality is reduced and the degree of quality reduction is dependent on environmental factors, with high rainfall having a large influence on this reduction (Ellery et al., 1995). Again, this implies that management for forage quality should vary between the growing season and the dormant season and should vary depending on climate, environmental variables, and livestock requirements.

The Serengeti migratory system is an example of a natural system adapted to forage quality and quantity provision throughout the year for large numbers of grazing animals (Murray and Illius, 1996). During the wet season, migratory animals are concentrated in the short-grass plains in the Southeast region of the Serengeti, where they graze short, actively growing green grass (grazing nonselectively at a relatively high density) during a time of high forage quality demand for lactation, young animal growth, and conditioning males and breeding females for the imminent breeding season (McNaughton, 1985). During this period, animals move around, keeping the grass short and actively growing (Hopcraft et al., 2014). During the dry season, the migratory animals congregate in the Northwest regions where much of the forage has grown unchecked during the wet season and is available as a high biomass/low-quality forage during a time of reduced forage quality demand. Here, animals graze the standing biomass at a relatively lower density with a higher degree of selectivity (McNaughton, 1985), with regrowth commencing during the wet season after the migratory animals have moved on. In the Serengeti, there are many grazing animals that do not migrate, including buffalo. Typically, these move from high catenal positions (short, high-quality grass) during the wet season to lower positions during the dry season (taller grass grown out during the wet season) in what has been described as a “mini-migration” (Hopcraft et al., 2014).

Another relevant example from unmanaged natural systems is the grazing patterns of the white rhinoceros. During the growing season, white rhino grazing is usually confined to grazing lawns, being patches of very short grass. They maintain these short, actively growing, high-quality forage patches by repeated grazing for the duration of the wet season (Owen-Smith, 1988). During the dormant season, the white rhinos move to areas of taller grass that have grown relatively unchecked during the growing season, where they trade-off quality for quantity (Owen-Smith, 1988).

Parallels between the Serengeti migratory system, the “mini-migration” in the Serengeti and white rhino grazing patterns are obvious, where the focus is on wet season quality and dry season quantity. Within the wet season, movement is focused on ensuring enough quantity of high-quality forage, with repeated grazing ensuring an adequate amount of short, actively growing grass. The term “surfing the green wave” is an elegant description of this phenomenon (Middleton et al., 2018). Within the dry season, movement is focused on accessing enough quantity. Without regrowth during the dry season, movement is likely to be sequential i.e., an area grazed once before animals move on. These examples of natural systems have strong parallels with the flexible grazing management system proposed by Venter and Drewes (1969).

### Fire and grazing interaction

In the absence of fire in mesic grasslands, grazing tends to become more selective over time, due to the inherent differences in palatability and acceptability between grass species. Periodic fire serves to remove all accumulated unpalatable forage, and resets the grazing selectivity patterns, as most grass species tend to be acceptable after fire (Briske, 1996). In addition, mesic grasslands are generally highly adapted to fire and regular fire usually has a positive impact on species richness and diversity (Fuhlendorf et al., 2017).

Fire, in mesic grasslands, increases forage quality and animal performance, particularly in the early part of the growing season, while potentially reducing above ground net primary productivity over the same period. This can present a trade-off between quality and quantity, however it is likely that the reduced biomass will be offset by the increased spectrum of species being grazed after fire (Mentis and Tainton, 1984) with associated advantages for maintaining cover of palatable grasses when unpalatable neighbors are grazed. Incorporation of fire into a grazing management system should thus depend on the objectives of the grazing system, the potential consequences of either including or excluding fire, and importantly, post fire management.

### Philosophy and principles

The first and most crucial step in grassland and savanna management for livestock production is to set aside a large enough area of grassland for season-long resting to produce adequate forage for winter, which also provides a whole growing season for recovery of grasses after grazing for sustainability purposes (maintaining high-quality grassland). The second step is to identify the growing season area and develop grazing plans for the growing season as well as plans for physical separation of the areas (i.e., keep animals out of the winter area during summer). In the higher rainfall areas where the rainy season is around six months, the summer and winter grazing areas are likely to be similar in size.

During the summer, if, for example, animals are grazing on half of the available area, this results in an automatic doubling of the density relative to the stocking rate. Additional subdivision can serve to increase the density further if required, bearing in mind that maintaining a short, actively growing grass sward will enhance quality and animal performance. During the winter, density becomes less important although subdivisions allow for effective rationing of forage for the winter period and may alleviate chronic shortages towards the end of winter.

Total animal numbers, or stocking rate, remain important in the relationship between forage production and forage consumption. The suggested approach provides a useful means of assessing this relationship in the manner of an early warning system. If, in a normal rainfall year, forage becomes depleted prior to the planned move to winter or summer grazing area, then forage consumption is higher than production, and animal numbers should be reduced accordingly before forage shortages impact livestock production. If there is excess forage at the time of moving to the winter or summer area, then forage production exceeds consumption, and animal numbers could be increased. This relationship between forage production and consumption is likely to vary from year to year based on rainfall. In years of excess production, fire could be incorporated in the management, with consequent improvement of forage quality, reduction of shrub and bush encroachment and benefits to plant species that are dependent on fire. In years where consumption equals or exceeds production, there will be reduced need for fire. During years of forage shortage, fire consumes plant biomass that could be grazed and should be avoided.

### Management practices

It is unlikely that any management approach or system is adopted on-farm entirely from research results. In reality, principles adopted from research are commonly combined with practical experience to develop grazing management approaches applied by livestock managers (Stuart-Hill, 1989). This is likely to exacerbate the lack of clear differentiation in the outcomes between various grazing management approaches when assessing impacts at farm scale.

Local circumstances influence management practices. For example, in Australia the large ranch and paddock sizes, coupled with high costs of labor preclude most forms of management intensification. This results in a strong focus on stocking rate as the main management factor under control of the manager, and manipulating stock numbers depending on rainfall (O’Reagain et al., 2014). While wet season resting is strongly promoted, it is not widely practiced. Nonetheless, wet season spelling requires low management intervention, and provides significant benefits.

#### Wet season grazing management

**Figure 5.**
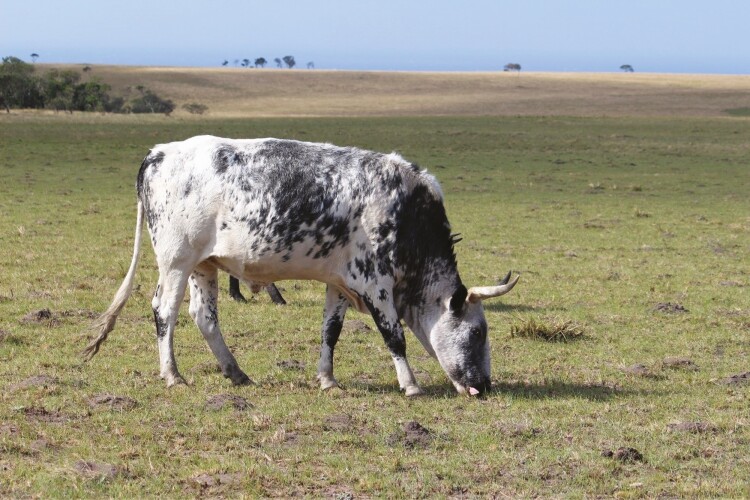
Example of short, high-quality grazing following fire in a mesic grassland. Photo by K P Kirkman.

In practice, a combination of judiciously timed fire (depending on rainfall) with confinement by herding or subdivision is likely to be the most viable option for providing optimal quality and quantity of forage, while minimizing grazing selectivity during the growing season (Figure 5).

Maintaining short, actively growing grass to optimize quality requires relatively high grazing pressure. If or when animals are moved to fresh grazing, it is important to move back to the first grazed area before it gets too tall, so that quality remains high. This implies a strategically, adaptively managed, irregular grazing cycle in the growing season, with a gradient from intensively grazed short, high-quality grass (priority planned high-quality grazing area) to taller, lower quality, less intensively grazed grass (reserve grazing area). In seasons of below average rainfall, there should be little or no areas that grow tall and lose quality. In seasons of high rainfall, there may be significant areas that grow tall and lose quality. This should not affect animal performance if grazing is concentrated on the short, high-quality (priority) areas.

#### Dry season grazing management

During the dry season, there is no regrowth following grazing. Quality is likely to decline gradually throughout the dry season, following the normal senescence patterns of the grass. Under these circumstances the most logical approach is to move animals systematically through the dry season grazing area, managing for quantity (intake) and not quality. The ability to store sufficient forage for the dry season will depend on how much area is rested during the growing season, which will also be influenced by stocking rate.

#### Examples

Long-term monitoring of rangeland condition on several ranches of the Ghanzi region of Botswana (mean annual rainfall ~395 mm) show that seasonal resting and grazing of grassland as outlined above can result in an increase in the most desirable high-quality grasses over time, only if they occur above a specific abundance threshold where selective grazing is minimal (R. Fynn, unpublished data). . The findings show that when the abundances of the high-quality grasses are low (<20% to 40%) then they are selectively grazed giving taller, lower quality grasses a competitive advantage, which increase at the expense of the high-quality grasses. These results demonstrate that a priority paddock approach is required to rehabilitate grassland where the high-quality grass species occur at low abundance. The priority paddock approach of burning a paddock at the start of the growing season and then ensuring it is kept short all season will ensure that the shorter grasses are not selectively grazed and thereby ensure that the taller grasses do not get a competitive advantage. The priority paddock approach, as described for the Venter–Drewes system, has been demonstrated in the Dundee region of KwaZulu-Natal, South Africa (mean annual rainfall ~840 mm) to result in a large increase in short, high-quality grasses and a decrease in taller grass species, while facilitating improved livestock performance at greater stocking rates.

## Conclusions

**Figure 6.**
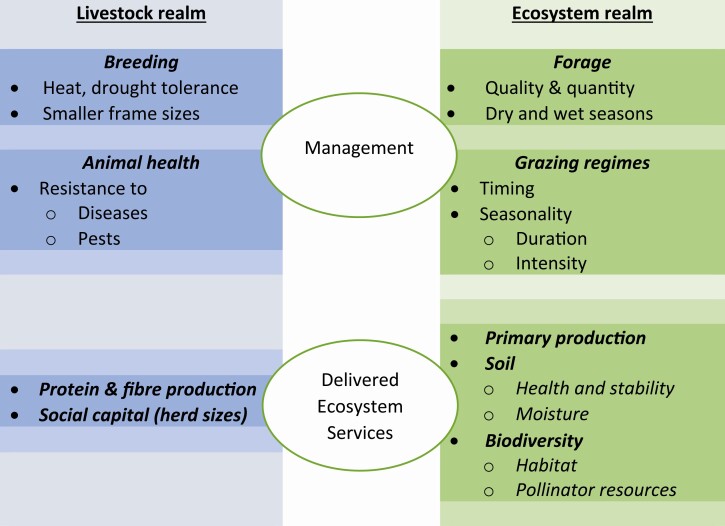
Schematic outline of grassland and livestock management pointers for ecologically and economically sustainable extensive livestock production from grasslands and savannas.

The grazing management principles outlined above closely resemble several natural wildlife systems, with adaptations for different livestock farming scenarios (Figure 6). The principles can be applied across commercial (fencing) and communal (herding) grazing systems, with unique adaptations under different scenarios. It is envisaged that effective grazing management (feeding), along with using adapted animals (breeding) should comprise the primary strategy for future-proofing livestock production in the face of climate change. Once the primary strategy is in place, secondary strategies comprising animal health and veterinary programmes and targeted supplementary feeding should receive focus.

*Conflict of interest statement.* None declared.

## About the Author(s)

**Kevin Kirkman** is a Professor of Grassland Science in the School of Life Sciences at the University of KwaZulu-Natal in South Africa. He received a BSc Agric and MSc Agric in Grassland Science and a PhD from the University of Natal. He has worked as a researcher for the Department of Agriculture in South Africa, followed by an advisory position in agricultural industry before joining the University of KwaZulu-Natal. His research interests include the ecology and management of grasslands and savannas, with a focus on fire and grazing ecology, as well as restoration of degraded grasslands. He is interested in developing and promoting grassland and savanna management strategies for livestock production while maintaining or improving grassland and savanna condition.

**Richard Fynn** is an associate professor of rangeland ecology at the Okavango Research Institute in Maun, Botswana. He received BSc degree in Botany and Grassland Science and a BSc (Hons) in Grassland Science from the University of Natal and an MSc and a PhD in in Grassland Science from the University of KwaZulu-Natal (UKZN). He did Post Docs working on vegetation ecology at UKZN and on plant-herbivore-fire interactions at Konza Prairie Biological Station, Kruger National Park and Ukulinga long-term burning experiments. His research interests include understanding functional habitat heterogeneity for large herbivores in African savannas, rangeland management strategies for sustainable and profitable cattle ranches and developing equitable and effective conservation strategies through a social-ecological systems approach.

**Devan Allen McGranahan** is a Rangeland Research Ecologist with the U. S. Department of Agriculture’s Agricultural Research Service in Miles City, Montana. After receiving a BA in Biology from Grinnell College, he spent a year in southern Africa studying the game ranching industry on commercial farms. He earned an MS in Sustainable Agriculture and PhD in Ecology and Evolutionary Biology from Iowa State University and spent time in the Department of Grassland Science at the University of KwaZulu-Natal as a Fulbright Scholar. He is the lead author of “Ecology of Fire-Dependent Ecosystems: Wildland Fire Science, Policy, and Management” (Taylor & Francis/CRC Press). Dr McGranahan’s research focuses on the integration of prescribed fire into extensive livestock production systems to simultaneously support the conservation of rangeland biodiversity, sustainable livestock production, and diverse rural livelihoods.

**Peter O’Reagain** is a research scientist with the Queensland Department of Agriculture and Fisheries in Charters Towers, Queensland, Australia. He received a BSc (Agric) from the University of Natal, MSc in Ecology and PhD from the University of the Witwatersrand in Johannesburg, South Africa. His research interests focus on sustainable grazing management for the livestock industry. He has been responsible for long-term Wambiana grazing experiments, initiated in 1997.

**Trevor Dugmore** graduated with a BSc and MSc in Animal Science from the University of Natal and a PhD from the University of KwaZulu-Natal. He commenced his career specializing in the feeding and management of dairy cattle on pasture and the nutritive evaluation of roughages at the Cedara Agricultural College and Research Station in the Department of Agriculture, progressing to Scientific Manager, Livestock Production Research with the KwaZulu-Natal Department of Agriculture and Rural Development. Trevor served as the scientific editor of the production manual, Feeding and Management of Dairy Cattle in KwaZulu-Natal, published by the KwaZulu-Natal Department of Agriculture and in various working groups on dairying in South Africa prior to his retirement. He is a subeditor for the South African Journal of Animal Science and the current President of the South African Society for Animal Science. Trevor is currently a Trustee and Board Member of the Mdukatshani Rural Development Trust, an NGO working in rural KwaZulu-Natal.
